# Establishment of a Triplex qPCR Assay for Differentiating Highly Virulent Genotype I Recombinant Virus From Low-Virulence Genotype I and Genotype II African Swine Fever Viruses Circulating in China

**DOI:** 10.1155/2024/6206857

**Published:** 2024-09-24

**Authors:** Leilei Ding, Tao Ren, Guoxia Bing, Zhigang Wang, Baoyue Wang, Jianqiang Ni, Yuliang Liu, Rui Zhao, Yuanmao Zhu, Fang Li, Renqiang Liu, Qiang Fu, Zhijun Tian, Zhigao Bu, Encheng Sun, Dongming Zhao

**Affiliations:** ^1^ State Key Laboratory for Animal Disease Control and Prevention National High Containment Facilities for Animal Disease Control and Prevention National African Swine Fever Para-reference Laboratory Harbin Veterinary Research Institute Chinese Academy of Agricultural Sciences, Harbin 150069, China; ^2^ College of Veterinary Medicine Xinjiang Agricultural University, Urumqi 830052, China; ^3^ China Animal Disease Control Center, Beijing 100125, China; ^4^ Institute of Western Agriculture The Chinese Academy of Agricultural Sciences, Changji 831100, China

**Keywords:** African swine fever virus, differential detection, genotype II, highly virulent genotype I, low virulent genotype I, triplex real-time quantitative PCR

## Abstract

African swine fever virus (ASFV) poses serious threats to the global swine industry, food safety, and the economy. Since August 2018, different types of ASFVs have successively emerged in China, making ASF diagnostics more challenging. The highly virulent genotype I recombinant virus has gradually become the prevalent dominant strain and is identified by sequencing several of its genes, which is time-consuming and expensive. Here, we developed a triplex real-time quantitative PCR (qPCR) assay based on the ASFV B646L, X64R, and MGF_360-14L genes to differentiate highly virulent genotype I recombinant viruses from low-virulence genotype I and genotype II viruses in China. This method has high sensitivity and a limit of detection of 10 copies/reaction for standard plasmids, as well as good specificity without cross-reactions with the viral nucleic acids of porcine reproductive and respiratory syndrome virus (PRRSV), classical swine fever virus (CSFV), pseudorabies virus (PRV), porcine circovirus 2 (PCV 2), porcine circovirus 3 (PCV 3), porcine epidemic diarrhea virus (PEDV), transmissible gastroenteritis virus (TGEV), or porcine rotavirus (PoRV). Importantly, triplex qPCR can be used to quickly and accurately evaluate clinical samples and cell cultures infected with highly virulent genotype I virus, low-virulence genotype I virus, or genotype II virus. Thus, triplex qPCR provides an alternative tool for ASF surveillance in China.

## 1. Introduction

African swine fever (ASF) is an acute, contagious disease caused by ASF virus (ASFV). ASFV is an enveloped, double-stranded DNA virus with a 170–190 kb genome that encodes 151–167 proteins [1]. ASFVs can be divided into highly virulent, moderately virulent, and low virulent strains on the basis of their virulence in swine. According to sequence variations among the 478 nt at the C-terminus of the B646L gene (which encodes the p72 protein), ASFVs have been divided into at least 24 genotypes, which are all present in Africa, whereas only genotypes I and II have spread outside of Africa [2]. A recent study revealed that ASFVs can be divided into only six genotypes on the basis of their p72 protein sequences [3].

ASF has led to the death of more than 1.35 million pigs in 50 countries across Africa, Europe, Asia, and the Americas from January 2022 to January 29, 2024, causing substantial economic losses [4]. In August 2018, ASF was first reported in China; it was caused by the highly virulent genotype II Georgia07-like ASFV and quickly swept across the whole country in a few months [5]. Pigs infected with the virus show acute clinical symptoms of high fever, depression, hemorrhage, cyanosis, and death [5]. In 2020, the lower virulent genotype II viruses were detected in China, which had no hemadsorption (non-HAD) due to disrupted expression of their CD2v proteins and caused subacute and chronic disease with a longer disease course and lower mortality in pigs [6]. In 2021, low-virulence genotype I NH/P68-like ASFVs, which are also non-HADs and cause chronic disease signs, including intermittent fever, weight loss, chronic skin ulcers, and arthritis in pigs, were reported in China [7].

Most recently, highly virulent genotype I recombinant ASFVs emerged in the Jiangsu, Henan, and Inner Mongolia provinces of China, with more than 56% of their genomes containing 10 discrete fragments derived from genotype II Georgia07-like virus and showing similar virulence to that of HLJ/18 in pigs [8]. Importantly, the live attenuated vaccine derived from genotype II ASFV (HLJ/18-7GD) does not provide cross-protection against challenges with the highly virulent genotype I recombinant ASFV [8]. Although the highly virulent genotype II ASFV is still the dominant strain, the prevalence of highly virulent genotype I recombinant ASFV is gradually increasing in China [9]. Currently, highly virulent genotype I recombinant ASFV is identified by sequencing several different viral genes, including the B646L, EP402R, and MGF360-505 genes, which are time-consuming and expensive. Therefore, a detection method that easily differentiates highly virulent genotype I recombinant ASFV from genotype II virus and low-virulence genotype I virus with high sensitivity and stability is urgently needed to help prevent and control ASF in China.

Here, we developed a triplex qPCR assay based on the B646L, X64R, and MGF_360-14 L genes of ASFV that differentiates between the highly virulent genotype I recombinant virus and the low-virulence genotype I and genotype II viruses and evaluated its analytical sensitivity, analytical specificity, and repeatability.

## 2. Materials and Methods

### 2.1. Viruses and Viral Nucleic Acids

Highly virulent genotype I recombinant viruses (JS/LG/21, HeN/123014/22, and IM/DQDM/22), low-virulence genotype I viruses (SD/DY-I/21 and HeN/ZZ-P1/21), the highly virulent genotype II virus (HLJ/18), and the lower virulent genotype II virus (HLJ/HRB1/20) were isolated from clinical samples in the field in China [5–8]. The cDNA/DNA of classical swine fever virus (CSFV), porcine reproductive and respiratory syndrome virus (PRRSV), porcine epidemic diarrhea virus (PEDV), pseudorabies virus (PRV), porcine transmissible gastroenteritis virus (TGEV), porcine rotavirus (PoRV), porcine circovirus 2 (PCV 2), and porcine circovirus 3 (PCV 3) were extracted from virus cultures, which were stored at the Harbin Veterinary Research Institute, Chinese Academy of Agricultural Sciences, and stored at −70°C in our laboratory.

### 2.2. Standard Plasmids

The X64R and MGF_360-14 L genes were amplified from JS/LG/21 genomic DNA and cloned and inserted into the pCAGGS-MCS vector (named pCAGGS-X64R and pCAGGS-MGF) as standard plasmids, respectively. The following PCR primers were designed: X64R-F: 5′ (*SacI*) - C*GAGCTC*G TGG TTT GCT GAC TAT TTG GAA - 3′; X64R-R: 5′ (*XhoI*) - CC*CTCGAG*GG GCG TTT TCA AAG CAT ATA AAG GAT - 3′; MGF-F: 5′ (*SacI*) - C*GAGCTC*G TAA TAA CGC TAG AAG GCT TGT TT - 3′; and MGF-R: 5′ (*XhoI*) - CC*CTCGAG*GG TGG GCT TTA TAG TCC TTT GC - 3′. The standard plasmid of the B646L gene, named pCAGGS-B646L, was constructed in our laboratory [10] and stored at −20°C.

### 2.3. Viral Genomic DNA Extraction

The samples, including the tissue, swabs, EDTA-blood, and cell culture samples, were processed as previously described [5, 7, 8], and ASFV genomic DNA was extracted via the TIANamp Genomic DNA Kit (TIANGEN, Beijing, China; Cat: DP304-03) according to the manufacturer's instructions. All nucleic acid samples were stored at −20°C.

### 2.4. Primers and Probes for Triplex qPCR

A total of 156 whole genome sequences of ASFVs, including 74 genotype I and 82 genotype II ASFVs, were retrieved from the GenBank database, and their information is shown in Table S1. These sequences were analyzed via ClustalW via MegAlign software (DNASTAR) and Snapgene 4.1.8 software. On the basis of the unique and highly conserved regions of the X64R gene from the genotype I ASFVs, the MGF_360-14 L gene from the highly virulent genotype I and genotype II ASFVs, and the B646L gene from the genotype I and II ASFVs, three pairs of specific primers and probes were designed via Primer Express 3.0 software (Applied Biosystems, Foster City, California, USA). The three probes for the X64R, B646L, and MGF_360-14 L genes were labeled with VIC, FAM, and CY5, respectively, at the 5′ ends. All the primers and probes were synthesized by Sangon Biotech Co. Ltd. (Shanghai, China).

### 2.5. Development and Optimization of Triplex qPCR

To preliminarily test the triplex qPCR method, ASFV genomic DNA of the highly virulent genotype I recombinant virus (JS/LG/21), the low virulent genotype I virus (SD/DY-I/21), the highly virulent genotype II virus (HLJ/18), and the lower virulent genotype II virus (HLJ/HRB1/20) served as templates to detect the X64R, MGF_360-14 L, and B646L genes, respectively.

As previously described [11], the reaction conditions, including the primer and probe concentrations and cycling conditions, were optimized for triplex qPCR. The reaction system volume was 25 μl: 12.5 μl of 2 × Premix Ex Taq (TaKaRa, China), 0.5 μl of each primer (10 μM), 0.5 μl of each probe (10 μM), 5 μl of template DNA, and 3 μl of ddH_2_O. Triplex qPCR was performed with a Bio-Rad CFX96 Touch Real-Time PCR Detection System under the following reaction conditions: 95°C for 30 s, followed by 40 cycles of 95°C for 10 s and 59°C for 20 s. Fluorescence was recorded at 59°C.

### 2.6. Standard Curves and Analytical Sensitivity of Triplex qPCR

The standard plasmids pCAGGS-X64R, pCAGGS-B646L, and pCAGGS-MGF were extracted via the E.Z.N.A. Plasmid DNA Mini Kit I (OMEGA, USA). Their concentrations were detected via a Thermo Nanodrop Lite apparatus (Thermo Fisher Scientific, USA) and were converted into copy numbers via the following formula: *y* (copies/μl) =  (6.02 × 10^23^) × (*x*(ng/μl) × 10^−9^ DNA)/(DNA length × 660), which yielded 10^11^ copies/5 μl. The three standard plasmids were subsequently mixed such that the concentration of each plasmid was 10^10^ copies/5 μl. Finally, tenfold dilutions of the standard plasmids from 10^6^ to 10^1^ copies were prepared and used to generate standard curves to assess sensitivity. Each sample was tested in triplicate in an experiment.

### 2.7. Analytical Specificity of the Triplex qPCR

The genomic cDNA or DNA of PRRSV, CSFV, PRV, PCV 2, PCV 3, PEDV, TGEV, PoRV, and ASFVs (JS/LG/21, SD/DY-I/21, and HLJ/18), whose viral titers were greater than 10^6^ TCID_50_, were used to evaluate the specificity of triplex qPCR. Each virus sample was tested in triplicate.

### 2.8. Repeatability Analysis of the Triplex qPCR

The mixtures of standard plasmids bearing the X64R, B646L, and MGF_360-14 L genes at 10^6^, 10^5^, 10^4^, 10^3^, 10^2^, and 10^1^ copies/5 μl were detected via triplex qPCR to assess their repeatability (intra-assay and inter-assay precision). As previously described [12], for intra-assay variability, each dilution of the plasmid mixture was tested in triplicate on the same day, whereas for inter-assay variability, each dilution of the standard plasmids was tested in six independent experiments performed by two operators on different days. The coefficients of variation (CVs) of the Ct values were calculated on the basis of the intra-assay and inter-assay results.

### 2.9. Comparison of the Triplex qPCR With the qPCR Developed by Fernández-Pinero et al. [13]

ASFV DNA was extracted from clinical samples, including two samples of EDTA-blood, oral, rectal, lung, spleen, and lymph node samples from pigs infected with JS/LG/21 (highly virulent genotype I), SD/DY-I/21 (low virulent genotype I), or HLJ/18 (highly virulent genotype II) virus strains [5, 7, 8], respectively. The samples were processed as follows: the swabs were resuspended in 1 ml of PBS containing 1% penicillin and streptomycin and centrifuged at 8000 × *g* and 4°C for 5 min. Tissues (0.2 g) were homogenized in 1 ml of PBS containing 1% penicillin and streptomycin and centrifuged at 8000 × *g* and 4°C for 5 min. Blood was collected with EDTA as an anticoagulant and mixed thoroughly. Two hundred microliters of each sample were used to extract nucleic acid via the TIANamp Genomic DNA Kit. In addition, viral DNA was extracted from cultures of SD/DY-I/21, HeN/ZZ-P1/21 (low virulent genotype I), JS/LG/21, HeN/123014/22 (highly virulent genotype I), IM/DQDM/22 (highly virulent genotype I), HLJ/18, and HLJ/HRB1/20 (lower virulent genotype II) viruses. Because the triplex qPCR and the qPCR developed by Fernández-Pinero et al. [13] have the same target (B646L gene) and Ct value (40) of the adjudication results (positive or negative), all the DNA samples were then tested via both methods. The qPCR method developed by Fernández-Pinero et al. [13] is also known as UPL, which uses the following forward primer (ASF-VP72-F): 5′-CCC-AGG-RGA-TAA-AAT-GAC-TG-3′; reverse primer (ASF-VP72-R): 5′-CAC-TRG-TTC-CCT-CCA-CCG-ATA-3′; and probe: (5′-[6-carboxyfluorescein (FAM)]-TCC-TGG-CCR-ACC-AAG-TGC-TT-3′-[black hole quencher (BHQ)]), as previously described. Ultimately, the samples with Ct < 30 were sequenced to confirm the accuracy of the genotype classification.

## 3. Results

### 3.1. Design of Primers and Probes for Triplex qPCR to Distinguish Highly Virulent Genotype I From Low- Virulent Genotype I and Genotype II ASFVs

**Figure 1:**
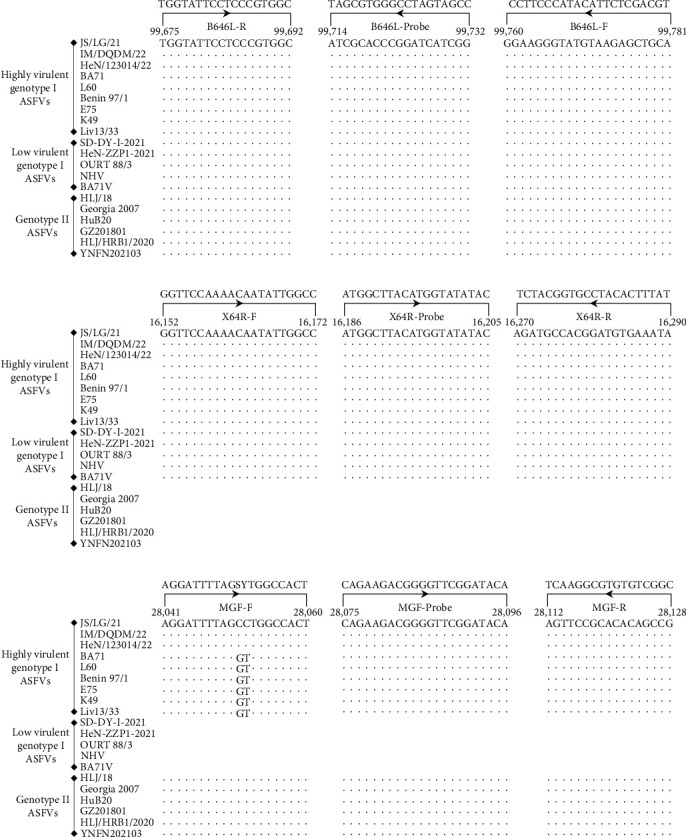
Sequences and locations of the primers and probes used for triplex qPCR. The nucleotide sequences of nine highly virulent genotype I ASFVs, five low-virulence genotype I ASFVs, and six genotype II ASFVs are presented to show the primers and probes specific for the B646L gene (A), the X64R gene (B), and the MGF_360-14 L gene (C). The positions of the primers and probes are indicated by the locations in the genome of ASFV JS/LG/21. Dots (.) indicate identical bases. Gaps represent gene deletions in the viral genome. F and R indicate the forward primer and reverse primer, respectively. MGF, the MGF_360-14 L gene. S, C/G bases and Y, C/T bases in the MGF-F primer.

Genome sequence analysis revealed that all genotype I viruses have the X64R gene (named the J64R gene for BA71 [14], BA71V [14], K49 [15], and Liv13/33 [16]), whereas all genotype II viruses lack this gene. The MGF_360-14 L gene was found in the genomes of the highly virulent genotype I and II ASFVs, whereas all low-virulence genotype I viruses lacked this gene. The B646L gene is extensively used as a target gene for ASF diagnosis, as recommended by the WOAH [13, 17, 18]. The conserved sequences of the X64R, MGF_360-14 L, and B646L genes from genotype I and/or genotype II ASFVs were selected to design primers and probes for differential detection of the highly virulent genotype I virus, the low-virulence genotype I virus, and the genotype II virus (Figure 1A–C). The three probes for the B646L, X64R, and MGF_360-14 L genes were labeled with the FAM, VIC, and CY5 fluorophores, respectively.

### 3.2. Development of Triplex qPCR

**Figure 2:**
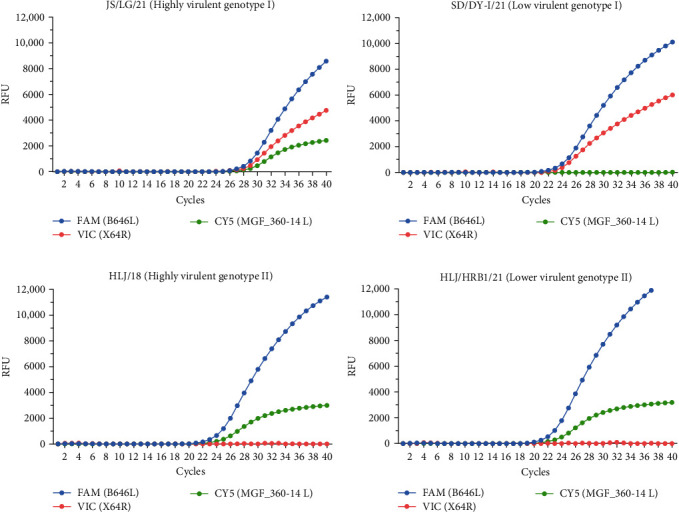
Establishment of triplex qPCR. The fluorescence signals of (A) FAM, VIC, and CY5 for the highly virulent genotype I virus JS/LG/21; (B) of FAM and VIC for the low virulent genotype I virus SD/DY-I/21; and of FAM and CY5 for (C) the highly virulent genotype II virus HLJ/18 and (D) the lower virulent genotype II virus HLJ/HRB1/20 were monitored via triplex qPCR. No fluorescent signal was detected from the negative control (ddH_2_O, not shown in the diagram). The RFUs of different cycles are expressed as the means of three replicates from a sample in an experiment.

For the SD/DY-I/21 DNA, the FAM (B646L) and VIC (X64R) fluorophores were detected; for the DNA of HLJ/18 and HLJ/HRB1/20, the FAM (B646L) and CY5 (MGF_360-14 L) fluorophores were detected; and for the JS/LG/21 DNA, the three fluorophores of FAM (B646L), VIC (X64R), and CY5 (MGF_360-14 L) were detected (Figure 2). The primer and probe concentrations and cycling conditions were optimized as described in Section 2.4.

### 3.3. Standard Curves and Analytical Sensitivity of Triplex qPCR

**Figure 3:**
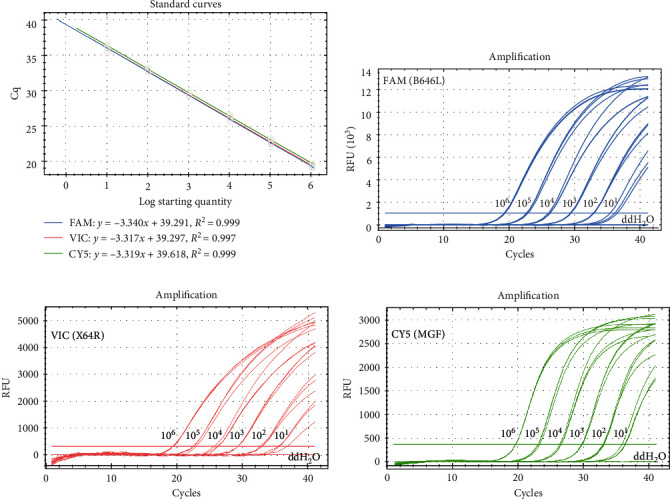
Standard curves and sensitivity of triplex qPCR. Tenfold serial dilutions ranging from 10^6^ to 10^1^ copies/5 μl of the standard plasmid mixture (pCAGGS-B646L, pCAGGS-X64R, and pCAGGS-MGF) were detected via triplex PCR. Each sample was tested in triplicate. (A) Standard curves and (B) sensitivity of the assay for the B646L gene. (C) Sensitivity of the assay for the X64R gene. (D) Sensitivity of the assay for the MGF_360-14 L (MGF) gene.

To assess the analytical sensitivity of triplex qPCR, tenfold serial dilutions of standard plasmid mixtures (pCAGGS-B646L, pCAGGS-X64R, and pCAGGS-MGF) ranging from 10^6^ to 10^1^ copies were detected via this method. The standard curves are shown in Figure 3A; the corresponding slopes of the equation, correlation coefficient (*R*^2^), and amplification efficiency (*E*) were −3.340%, 0.999%, and 99.3% for the B646L gene (FAM); −3.317%, 0.997%, and 100.2% for the X64R gene (VIC); and −3.319%, 0.999%, and 100.1% for the MGF_360-14 L gene (CY5). The limit of detection of triplex qPCR was 10 copies per reaction for all three genes (Figure 3B–D). These results indicate that the established triplex qPCR method has high efficiency, strong linear correlation, and good analytical sensitivity.

### 3.4. The Analytical Specificity of Triplex qPCR

**Figure 4:**
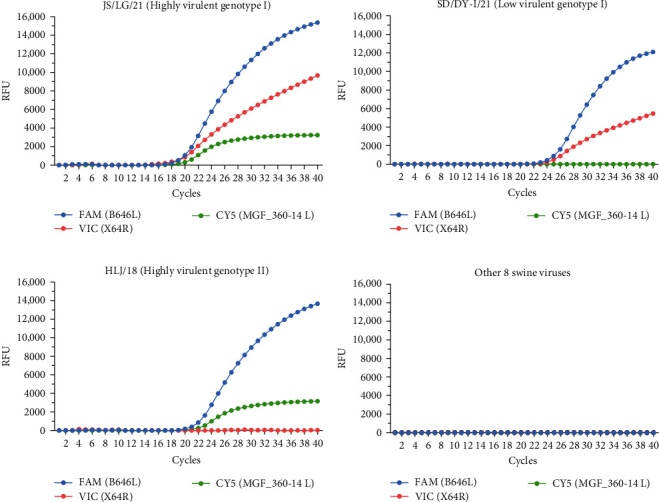
Specificity of triplex qPCR. The FAM, VIC, and CY5 fluorescence signals were detected via triplex qPCR; (A) viral DNA of the highly virulent genotype I virus JS/LG/21, (B) the low virulent genotype I virus SD/DY-I/21, and (C) the genotype II virus HLJ/18 served as positive controls. (D) No fluorescence signal was detected when the viral genomes of PRV, PRRSV, CSFV, PEDV, PCV 2, PCV 3, TGEV, and PoRV or the negative control (ddH_2_O) were used as templates. The RFUs of different cycles are expressed as the means of three replicates from a sample in an experiment.

To evaluate the analytical specificity of triplex qPCR, nucleic acids extracted from ASFVs (JS/LG/21, SD/DY-I/21, and HLJ/18), PRRSV, CSFV, PRV, PCVs (PCV 2 and PCV 3), PEDV, TGEV, and PoRV were used as templates. As shown in Figure 4, only the ASFV DNA samples presented positive amplification as follows: FAM, VIC, and CY5 signals for JS/LG/21; FAM and VIC signals for SD/DY-I/21; and FAM and CY5 signals for HLJ/18. All other samples, including ddH_2_O, were not amplified (Figure 3). The results demonstrate that triplex qPCR has good analytical specificity and no cross-reactivity with eight other swine viruses when their nucleic acid is dissolved in ddH_2_O.

### 3.5. Repeatability Analysis of Triplex qPCR

To assess the repeatability of the triplex qPCR, standard plasmid mixtures (pCAGGS-B646L, pCAGGS-X64R, and pCAGGS-MGF) were evaluated at six different concentrations (10^6^−10^1^ copies). The results are shown in Table 1. The intra-assay CVs ranged from 0.14% to 2.02% for the B646L gene (FAM), 0.16%–1.59% for the X64R gene (VIC), and 0.11%–1.69% for the MGF_360-14 L gene (CY5). The inter-assay CVs ranged from 1.33% to 2.33% for the B646L gene (FAM), 1.91%–2.66% for the X64R gene (VIC), and 0.79%–2.32% for the MGF_360-14 L gene (CY5). These results showed that the CVs of the triplex were 0.11%–2.66%, which satisfies the repeatability of the real-time PCR assay.

### 3.6. Comparison of Triplex qPCR and the qPCR Method Developed by Fernández-Pinero et al. [13]

To further evaluate the reliability of triplex qPCR for ASF diagnosis, 43 samples, including EDTA-blood, oral and rectal swabs, tissues, and cell cultures from pigs or porcine alveolar macrophages (PAMs) infected with genotype I and II ASFVs, were tested via this method and the qPCR method developed by Fernández-Pinero et al. [13]. All 43 samples were detected as positive and differentiated by triplex qPCR, which included 15 samples of highly virulent genotype I ASFV, 14 samples of low virulent genotype I ASFV, and 14 samples of genotype II ASFV. The positive results were consistent with those detected by the qPCR method developed by Fernández-Pinero et al. [13] (Table 2). The B646L Ct values of the same positive sample were similar for both methods. These results showed that both the methods of triplex qPCR developed by us and the qPCR developed by Fernández-Pinero et al. [13] have comparable efficiencies. In addition, the discrimination results of the triplex qPCR were consistent with the sequencing results for samples with Ct < 30, demonstrating that the triplex qPCR results were reliable.

## 4. Discussion

In this study, we first analyzed 156 ASFV genomes from 74 genotype I and 82 genotype II ASFV strains and reported that the X64R gene is present and highly conserved only in highly and low-virulence genotype I ASFVs, as is the MGF_360-14 L gene in highly virulent genotype I and genotype II ASFVs. Accordingly, we established triplex qPCR targeting the ASFV X64R, MGF_360-14 L, and B646L genes that differentiate the highly virulent genotype I recombinant virus from the low-virulence genotype I and II ASFVs circulating in China.

The established triplex qPCR method provides fast, sensitive, specific, accurate, and reproducible detection of highly virulent genotype I recombinant viruses, low-virulence genotype I viruses, and genotype II ASFVs in the field in China. This method has good sensitivity, with a detection limit of 10 copies per reaction for the standard plasmids pCAGGS-X64R, pCAGGS-B646L, and pCAGGS-MGF, which is similar to the sensitivity of the single qPCR method developed by Fernández-Pinero et al. [13], which targets the ASFV B646L gene (<18 copies/reaction), and the duplex qPCR method, which targets the ASFV B646L gene (10 copies/reaction) [10] and the E296R gene (10 copies/reaction) [19], which are used for differential detection of genotype I and genotype II ASFVs. This approach is also similar to the sensitivity of triplex qPCR, which targets the ASFV B646L (7.9 copies/reaction), EP402R (9.6 copies/reaction), and MGF_360-14 L (9.7 copies/reaction) genes [20], which are used for the differentiation of wild-type ASFVs and gene-deleted strains. Moreover, the CVs of triplex qPCR were 0.11%–2.66%, which is comparable with those of the qPCR methods developed by Wang et al. [21] (the CVs ranged from 0.05% to 2.75%), Li et al. [19] (the CVs ranged from 0.23% to 3.49%), and Qian et al. [22] (the CVs ranged from 0.16% to 1.88%) in the repeatability evaluation.

The assay also shows high specificity, no cross-reactivity with eight other swine viruses (Figure 4), and good repeatability when dissolved in ddH_2_O (Table 1). Moreover, 43 samples, including EDTA-blood, oral and rectal swabs, tissues, and cell cultures from pigs or PAMs infected with different virulence genotype I and II ASFVs, were successfully detected and differentiated via this method (Table 2). Similarly, the mean Ct values of these samples for the B646L gene via both methods were 27.83 (triplex qPCR) and 28.53 (UPL). However, according to the WOAH validation methods, additional clinical samples, including negative blood, tissue homogenate, or other typical samples, are still needed for further validation.

MGF_360-14 L is a member of the multigene family MGF_360-505R, which includes important virulence-associated genes [23, 24]. Strains in which these genes are deleted, such as HLJ/18-7GD [25] and ASFV-G-*Δ*MGF [26], have become promising ASF attenuated vaccine candidates. Moreover, MGF_360-14 L is also a target gene used for the differential detection of ASFV gene-deleted vaccine and wild-type strains [16, 27]. Therefore, triplex qPCR can differentially detect genotype II ASFVs in which MGF_360-14 L is deleted. For these genotype II ASFV strains, the results of the assay showed positive amplification for only the B646L gene (FAM). However, this method cannot differentiate the highly virulent genotype II virus from the lower virulent genotype II virus, in which the CD2v proteins are truncated or not expressed [6]. For both virulent genotype II ASFVs, the results of the assay revealed positive amplification of the B646L (FAM) and MGF_360-14 L (CY5) genes. Further evaluation and validation are needed to determine whether our method could be used for other genotype ASFVs.

## 5. Conclusion

We developed a triplex qPCR method that effectively differentiates highly virulent genotype I recombinant viruses from low-virulence genotype I and genotype II ASFVs. The method has high sensitivity and specificity and can be used to detect viruses in clinical samples, including EDTA-containing blood, oral and rectal swabs, and tissues, thereby providing an alternative tool for ASFV surveillance in China.

## Figures and Tables

**Table 1:** Repeatability evaluation of triplex qPCR for intra-assay and inter-assay variation using a tenfold serial dilution of a standard plasmid mixture.

Standard plasmid mixture	The targeted genes	Concentration (copies/5 μl)	Ct values of intra-assay for repeatability ^*∗*^				Ct values of inter-assay for repeatability ^*∗*^			
			Assays run	$\overset{―}{X}$	SD	CV (%)	Assays run	$\overset{―}{X}$	SD	CV (%)
pCAGGS-X64R + pCAGGS-B646L + pCAGGS-MGF	X64R	10^6^	1	18.62	0.15	0.83	6	18.70	0.47	2.50
		10^5^	1	21.77	0.03	0.16	6	22.03	0.43	1.94
		10^4^	1	25.31	0.09	0.37	6	25.33	0.48	1.91
		10^3^	1	28.47	0.18	0.65	6	28.52	0.72	2.53
		10^2^	1	31.73	0.50	1.59	6	31.83	0.77	2.42
		10^1^	1	35.17	0.46	1.31	6	35.09	0.93	2.66
	B646L	10^6^	1	18.67	0.16	0.85	6	18.85	0.33	1.76
		10^5^	1	22.14	0.11	0.50	6	22.26	0.40	1.81
		10^4^	1	25.53	0.10	0.39	6	25.62	0.38	1.49
		10^3^	1	28.90	0.04	0.14	6	28.97	0.39	1.33
		10^2^	1	32.04	0.09	0.29	6	32.25	0.43	1.34
		10^1^	1	35.55	0.72	2.02	6	35.69	0.83	2.33
	MGF_360-14 L	10^6^	1	19.07	0.32	1.69	6	19.16	0.44	2.32
		10^5^	1	22.51	0.21	0.93	6	22.68	0.48	2.09
		10^4^	1	26.03	0.06	0.22	6	26.05	0.43	1.64
		10^3^	1	29.33	0.03	0.11	6	29.38	0.48	0.79
		10^2^	1	32.47	0.12	0.38	6	32.61	0.44	1.35
		10^1^	1	35.26	0.27	0.78	6	35.69	0.68	1.90

^*∗*^The repeatability is shown as the mean ± standard deviation (SD from three replicates).

**Table 2:** The 43 clinically positive ASFV samples were identified via triplex qPCR and UPL.

Types of ASFV	Types of clinical samples	Sample no.	The triple qPCR (Ct value)			The UPL (Ct value)
			X64R (VIC)	B646L (FAM)	MGF_360-14 L (CY5)	B646L (FAM)
Highly virulent genotype I ASFVs	EDTA-blood	1	25.14	26.25	24.62	25.48
		2	26.29	26.30	26.16	27.08
	Oral swab	3	27.45	28.58	28.00	29.27
		4	33.12	33.51	33.12	34.42
	Rectal swab	5	27.76	28.46	27.23	27.89
		6	30.69	31.35	31.25	32.31
	Lung	7	18.95	19.02	19.04	19.88
		8	21.96	21.97	21.88	22.97
	Speen	9	19.78	20.16	20.00	20.53
		10	23.14	24.19	23.89	24.50
	Lymph node	11	21.89	22.55	22.14	23.15
		12	21.83	22.07	22.01	23.10
	Cell culture (JS/LG/21)	13	15.64	15.41	15.61	16.23
	Cell culture (HeN/123014/22)	14	18.84	18.68	19.01	18.62
	Cell culture (IM/DQDM/22)	15	16.00	15.86	16.10	17.13

Low virulent genotype I ASFVs	EDTA-blood	16	31.21	31.23	−	31.79
		17	31.71	32.37	−	33.18
	Oral swab	18	32.12	33.03	−	33.18
		19	32.22	33.25	−	33.78
	Rectal swab	20	30.83	30.89	−	31.59
		21	31.27	32.15	−	32.74
	Lung	22	32.39	32.72	−	32.85
		23	31.59	32.78	−	33.75
	Speen	24	30.97	30.80	−	31.62
		25	31.43	32.13	−	32.83
	Lymph node	26	32.38	32.46	−	33.34
		27	32.47	33.32	−	33.73
	Cell culture (SD/DY-I/21)	28	28.70	28.70	−	27.35
	Cell culture (HeN/ZZ-P1/21)	29	27.97	27.97	−	27.53

Genotype II ASFVs	EDTA-blood	30	−	29.92	30.50	30.50
		31	−	26.30	27.06	26.98
	Oral swab	32	−	30.09	30.53	31.46
		33	−	32.06	32.30	33.26
	Rectal swab	34	−	33.50	33.56	34.01
		35	−	33.59	33.93	34.62
	Lung	36	−	29.66	30.25	30.17
		37	−	26.22	26.99	27.13
	Speen	38	−	29.87	30.25	32.18
		39	−	30.45	31.01	31.51
	Lymph node	40	−	29.27	29.91	31.41
		41	−	25.94	26.50	27.79
	Cell culture (HLJ/18)	42	−	20.89	21.01	22.02
	Cell culture (HLJ/HRB1/20)	43	−	20.72	20.94	21.95

Negative control	ddH_2_O	44	−	−	−	−

−, the result of qPCR was negative.

## Data Availability

The data that support the findings of this study are available from the corresponding author upon reasonable request.

## Supporting Information

Supporting Information Table S1: genotype I and genotype II ASFVs were used to analyze and design the primers and probes of the triple qPCR in this study.
