# Supplementary material for: Establishment of a Triplex qPCR Assay for Differentiating Highly Virulent Genotype I Recombinant Virus From Low-Virulence Genotype I and Genotype II African Swine Fever Viruses Circulating in China
Source: Transbound Emerg Dis. 2024 Sep 24;2024:6206857. doi: 10.1155/2024/6206857 (PMC12017118; doi:10.1155/2024/6206857)
Supplement: Supporting Information — Table S1: genotype I and genotype II ASFVs were used to analyze and design the primers and probes of the triple qPCR in this study. [file 6206857.f1.docx]

**Supplemental Materials**

**Table S1**. **Genotype I and genotype II ASFVs used to analyze and design primers and probes of triple qPCR in this study.**

| **Genotype** | **Virulence** | **No.** | **Strain name** | **Access No.** | **No.** | **Strain name** | **Access No.** | **No.** | **Strain name** | **Access No.** |
| --- | --- | --- | --- | --- | --- | --- | --- | --- | --- | --- |
| **I** | Highly | **1** | **Pig/Jiangsu/LG/2021** | **OQ504956** | **2** | **Benin 97/1** | **AM712239** | **3** | **Pig/Henan/123014/2022** | **OQ504954** |
|  |  | **4** | **BA71** | **KP055815** | **5** | **L60** | **KM262844** | **6** | **Pig/Inner Mongolia/DQDM/2022** | **OQ504955** |
|  |  | **7** | **E75** | **NC_044958** | **8** | **K49** | **MZ202520** | **9** | **Liv13/33 (OmLF2)** | **MN913970** |
|  | Low | **10** | **OURT 88/3** | **AM712240** | **11** | **NHV** | **KM262845** | **12** | **HeN-ZZP1-2021** | **MZ945536** |
|  |  | **13** | **SD-DY-I-2021** | **MZ945537** | **14** | **BA71V** | **NC_001659** | **/** | **/** | **/** |
|  | Unidentified in animals | 15 | 26544/OG10 | KM102979 | 16 | 47/Ss/2008 | KX354450 | 17 | 56/Ca/1978 | MN270969 |
|  |  | 18 | 57/Ca/1979 | MN270970 | 19 | 139/Nu/1981 | MN270971 | 20 | 140/Or/1985 | MN270972 |
|  |  | 21 | 85/Ca/1985 | MN270973 | 22 | 141/Nu/1990 | MN270974 | 23 | 142/Nu/1995 | MN270975 |
|  |  | 24 | 60/Nu/1997 | MN270976 | 25 | 26/Ss/2004 | MN270977 | 26 | 72407/Ss/2005 | MN270978 |
|  |  | 27 | 97/Ot/2012 | MN270979 | 28 | 22653/Ca/2014 | MN270980 | 29 | Ca1978_2 | MW723480 |
|  |  | 30 | Nu1979 | MW723481 | 31 | Nu1986 | MW723482 | 32 | Nu1990_1 | MW723483 |
|  |  | 33 | Nu1991_2 | MW723484 | 34 | Nu1991_3 | MW723485 | 35 | Nu1991_7 | MW723486 |
|  |  | 36 | Or1993_1 | MW723487 | 37 | Nu1993_2 | MW723488 | 38 | Nu1995_2 | MW723489 |
|  |  | 39 | Nu1995_3 | MW723490 | 40 | Nu1995_4 | MW723491 | 41 | 4996 WB | MW723492 |
|  |  | 42 | 46830 | MW723493 | 43 | 23221 | MW723494 | 44 | 72398 WB | MW723495 |
|  |  | 45 | 74377 | MW723496 | 46 | 22649 | MW723497 | 47 | 72912 WB | MW723498 |
|  |  | 48 | 22137 | MW723499 | 49 | 44076 | MW723500 | 50 | 47039 | MW736597 |
|  |  | 51 | 2019 WB | MW736598 | 52 | 98039 | MW736599 | 53 | 30322 | MW736600 |
|  |  | 54 | 49179 | MW736601 | 55 | 53706 | MW736602 | 56 | 63525 | MW736603 |
|  |  | 57 | 15998 | MW736604 | 58 | 51268 | MW736605 | 59 | 34403 | MW736606 |
|  |  | 60 | 32516 | MW736607 | 61 | 113049 WB | MW736608 | 62 | 6396 WB | MW736609 |
|  |  | 63 | 28928 | MW736610 | 64 | 56140 | MW736611 | 65 | 31208 | MW736612 |
|  |  | 66 | 33747 WB | MW736613 | 67 | 1537 WB | MW788405 | 68 | 22943_2008 | MW788406 |
|  |  | 69 | 31479_2005 | MW788407 | 70 | 35479_2014 | MW788408 | 71 | SS_1981 | MW788409 |
|  |  | 72 | 25185_2008 | MW788410 | 73 | Or_1984 | MW800838 | 74 | 7303WB | ON260839 |
| **II** | / | **1** | **Georgia 2007/1** | **FR682468** | 2 | Odintsovo_02/14 | KP843857 | 3 | Belgium 2018/1 | LR536725 |
|  |  | 4 | Moldova 2017/1 | LR722599 | 5 | CzechRepublic 2017/1 | LR722600 | 6 | Germany 2020/1 | LR899193 |
|  |  | 7 | Estonia 2014 | LS478113 | 8 | Pol16_20186_o7 | MG939583 | 9 | Pol16_20538_o9 | MG939584 |
|  |  | 10 | Pol16_20540_o10 | MG939585 | 11 | Pol16_29413_o23 | MG939586 | 12 | Pol17_03029_C201 | MG939587 |
|  |  | 13 | Pol17_04461_C210 | MG939588 | 14 | Pol17_05838_C220 | MG939589 | 15 | ASFV/POL/2015/Podlaskie | MH681419 |
|  |  | 16 | ASFV-SY18 | MH766894 | 17 | Georgia 2008/1 | MH910495 | 18 | China/2018/AnhuiXCGQ | MK128995 |
|  |  | **19** | **Pig/HLJ/2018** | **MK333180** | 20 | DB/LN/2018 | MK333181 | 21 | Belgium/Etalle/wb/2018 | MK543947 |
|  |  | 22 | ASFV/LT14/1490 | MK628478 | 23 | ASFV-wbBS01 | MK645909 | 24 | CN/2019/InnerMongolia-AES01 | MK940252 |
|  |  | 25 | ASFV Wuhan 2019-2 | MN393477 | 26 | ASFV Wuhan 2019-1 | MN393476 | 27 | ASFV/pig/China/CAS19-01/2019 | MN172368 |
|  |  | 28 | ASFV_HU_2018 | MN715134 | 29 | ASFV_NgheAn_2019 | MT180393 | 30 | ASFV/Kabardino-Balkaria 19/WB-964 | MT459800 |
|  |  | **31** | **GZ201801** | **MT496893** | 32 | ASFV/Korea/pig/PaJu1/2019 | MT748042 | 33 | Pol17_55892_C754 | MT847620 |
|  |  | 34 | Pol18_28298_O111 | MT847621 | 35 | Pol17_31177_O81 | MT847622 | 36 | Pol19_53050_C1959/19 | MT847623 |
|  |  | 37 | ASFV-wbShX01 | MW033528 | 38 | ASFV/Amur 19/WB-6905 | MW306190 | 39 | ASFV/Primorsky 19/WB-6723 | MW306191 |
|  |  | 40 | ASFV/Timor-Leste/2019/1 | MW396979 | 41 | China/GD/2019 | MW361944 | 42 | ASFV/Ulyanovsk 19/WB-5699 | MW306192 |
|  |  | 43 | ASFV2020-015-B | MW791755 | **44** | **HuB20** | **MW521382** | **45** | **Pig/Heilongjiang/HRB1/2020** | **MW656282** |
|  |  | 46 | ASFV2020-008-B | MW791752 | 47 | ASFV2020-014-B | MW791754 | 48 | VNUA-ASFV-05L1/HaNam/VN/2020 | MW465755 |
|  |  | 49 | ASFV2020-018-B | MW791756 | 50 | ASFV2020-019-B | MW791757 | 51 | ASFV2020-020-B | MW791758 |
|  |  | 52 | ASFV2020-021-B | MW791759 | 53 | ASFV2019-003-B | MW791760 | 54 | ASFV2020-003-B | MW791761 |
|  |  | 55 | MAL/19/Karonga | MW856068 | 56 | CADC_HN09 | MZ614662 | 57 | Odintsovo_02/14 | NC_044948 |
|  |  | 58 | ASFV Georgia 2007/1 | NC_044959 | 59 | HK_NT_202103 | OK358852 | 60 | Recombinant African swine fever virus | OL310288 |
|  |  | 61 | wild boar/SNJ/2020 | OL622042 | 62 | IND/AS/SD-02/2020 | OL692743 | 63 | IND/AR/SD-61/2020 | OL692744 |
|  |  | 64 | LYG18 | OM105586 | 65 | JX21 | OM105587 | 66 | SY-1 | OM161110 |
|  |  | 67 | GZ201801_2 | ON263123 | 68 | HB03A | ON380539 | 69 | HB31A | ON380540 |
|  |  | **70** | **YNFN202103** | **ON400500** | 71 | TAN/17/Kibaha | ON409979 | 72 | TAN/17/Mbagala | ON409982 |
|  |  | 73 | TAN/20/Morogoro | ON409983 | 74 | Yangzhou | ON456300 | 75 | A4 | ON963982 |
|  |  | 76 | ASF-MNG19 | OP467597 | 77 | SY-2 | OP612151 | 78 | Nigeria-RV502 | OP672342 |
|  |  | 79 | ASFV | OP823268 | 80 | ASFV | OP823269 | 81 | China/LN/2018/1 | OP856591 |
|  |  | 82 | ASFV JS | OR180113 | / | / | / | / | / | **/** |

Note: The black ASFV strains were showed in the Fig. 1-A, B, C. “/” represents all genotype II ASFV strains including highly, moderate or low virulent.
